# Consequences of load distribution in selected vehicles in the context of changing the position of the vehicle’s centre of gravity

**DOI:** 10.1038/s41598-023-48083-8

**Published:** 2023-11-29

**Authors:** František Synák, Alexandra Smolková, Klára Žúžiová

**Affiliations:** Department of Road and Urban Transport, University of Zilina, Univerzitná 1, 01001 Žilina, Slovakia

**Keywords:** Mechanical engineering, Applied physics

## Abstract

The position of the vehicle’s centre of gravity has an impact on its driving performances, and it also affects the vehicle’s impact on its surroundings. Therefore, the study pays attention to the change in the centre of gravity’s position due to different load distribution. The research was performed by wide-ranging experimental measurements with three different vehicles, i.e. with a passenger car, a van and a truck. The measurement results and their analysis point to the rate of change in the centre of gravity’s position due to different load distribution, and to the fact that a vehicle axle can be overloaded even when the vehicle load capacity is not used completely. In addition, the contribution of the study is a database which can be used for other research via modelling.

## Introduction

The centre of gravity is a point that includes the total weight of a vehicle. In a vertical plane, the position of the vehicle’s centre of gravity is defined by a height of its location from the road surface, in a longitudinal direction it is defined by its distance from the vehicle axles, and in a transverse direction it is defined by its distance from the vehicle edges^1,2^. The centre of gravity’s position is important when calculating the vehicle dynamics, and it affects the vehicle driving performances^3^. The distance between the centre of gravity and the axles are highly affected by their load. If the centre of gravity is too close to one of the axles, it may lead to its overload. The axle overload increases the risk of a sudden explosion of tyres, their wear and tear, and production of particulate matter from the tyres. It also leads to a change in car suspension characteristics and to an increase in wear and tear of the wheel suspension, damping, suspension system and the road surface. This can be concluded by the information from publications^4,5^. The issue of axle overload on the roads is also observed in publication^6^ in which the authors conclude that the axle load contributes to shortening the fatigue life of the pavement structure. The authors from publication^7^ introduce that the greatest difficulties in the area of damaging and degradation of the roads are the ruts as a cause of the axle overload with single tyres. Particulate matter from the tyres is also a problem since its emission is higher when the tyres are overloaded^8^. PM in the air of predominantly densely built-up cities is a serious issue and causes premature deaths^9,10^. There is also a danger of suspension destruction in connection with a sudden body descent that can be a reason for a car accident, as also given in publication^11^ in which the authors concluded that the axle road is the main cause of heavy weight vehicles’ malfunctions on highways in Poland, and then there is an increased risk of congestions due to obstructions, which is a frequent phenomena on the highways. Thus, the axle load has a negative impact on the economics, air quality as well as supply chains^12–14^. The axle load is also connected with a breach of legislation, and it could happen when the maximum permissible vehicle mass is not exceeded as well^15^. When the centre of gravity is too close to the rear axle, or behind it, too low load is on the front axle (the steering axle), and it has a negative impact on the vehicle’s ability to turn. Concerning a vehicle with the front driving axle, the ability to accelerate is reduced together with the front axle load since the value of transformed driving force from the wheels on the road depends on the friction coefficient and mass applied to a given axle. Thus, when reducing the vehicle’s ability to accelerate, the road safety is endangered, for example when driving from the side road to the main road and the junction throughput is also reduced since the vehicle needs more time to cross the junction^16,17^. Publication^18^ focused on the issues such as grip, braking and layout depending on the axle load. The authors observed a strong dependency between the centre of gravity’s position, and so the axle load, and the vehicle driving performances. The importance of the correct position of the centre of gravity within safe driving on the curved sections of mountain roads also implies from publication^19^ since the result of incorrect load distribution may be the vehicle to move too close to the outer side of the curve after a cut, for which reason the driver has to correct the trajectory, although overcorrection may move the vehicle into the oncoming lane. Publication^20^ observed the impact of the centre of gravity’s position on braking performances of van with a total mass of 3500 kg. The authors informed that increasing an inequality of the axle load and the centre of gravity’s position, with the same vehicle mass, braking deceleration decreases and braking distance lengthens. Such similar results are also shown in publication^21^ in which the measurements were performed with the vehicle weighing 18,000 kg. Assessing the impact of the centre of gravity’s position of tractor-semitrailer set on the braking safety indicators is given in publication^22^, and the authors observed a large impact of the centre of gravity’s position on braking performances as well. First, the centre of gravity’s position is given by the vehicle construction and its arrangement, in what way the engine, batteries, driving axles and so on are located^23^. Besides these, it is also strongly affected by the load mass and its distribution. Publication^24^ discusses the responsibility for the axle load due to incorrect load distribution relating to an automated vehicle as a response to a serious shortage of drivers on the roads. The centre of gravity’s position can also be changed when there is a loose cargo accompanied by intense braking, as given in publication^25^ as well. This cargo movement causes changes of a load of particular vehicle axles, which may result in overloading of axles mainly in a front part of the vehicle although the vehicle was loaded correctly^25^.

It follows from the literature overview that the axle load has an impact on road safety, vehicle dynamics, wear of tyres, other vehicle parts as well as roads, and on logistics, economics and air pollution. It can be concluded on the basis of the overview that major research, focused on the impact of load distribution on the vehicle centre of gravity’s position and vehicle dynamics, is conducted only via simulations which may cause various inaccuracies. Since the axle load is highly affected by load distribution in a vehicle, this study pays its attention therefore to the impact of load distribution on the axle load. The main research question is the extent to which the load distribution affects the axle load in relation to various types of vehicle, and also the risk of axle load when the load capacity is not used completely. The subsidiary research question is the possibility of using a common police axle scale for the measurements of axle load. Additionally, the objective of this publication is to increase the data on tractive force and the vehicle’s ability to accelerate depending on its axle load. In order to answer these questions and meet the objectives, the extensive experimental measurements have been performed. They were conducted under laboratory conditions via experimental measurements. The experimental measurements and the analysis of the values measured can answer the question whether the experimental measurements provide a substantial contribution in comparison with simulations with simple static analysis of a vehicle considered as a solid body that has a theoretical mass of which the centre of gravity’s position is estimated. To ensure the measurement comprehensiveness and its results, the measurements were performed together with three vehicles with different loads. The first vehicle was an estate passenger car. The second vehicle used for the measurements was a van with a maximum permissible weight of 3500 kg. The van was selected due to the fact that it is often used by the couriers and its high load capacity in comparison with the empty vehicle mass. The third vehicle was a truck with a maximum permissible weight of 18,000 kg.

The scientific and practical contribution of this article is the extensiveness and comprehensiveness of the measurements, and the result analysis which can further serve as a database as well as for other research workers focusing on the centre of gravity’s position, vehicle dynamics, road safety, accident reconstruction and the like. Compared to other publications, the advantage of this article lies in the way of determining the centre of gravity’s position by experimental measurements, not by the simulations. Experimental measurements cover all the factors affecting the centre of gravity’s position whereas when determining the position via mathematical simulations, it often leads to simplifications which can have an impact on accuracy of the calculations and adequacy of the following analyses. For instance, publication^26^ takes into consideration the fact during the mathematical modelling that the vehicle’s structure is assumed to be rigid, the vehicle is symmetric about its centreline, the lateral deflection of the suspension is negligible and the reaction forces from the road are applied at the centre of the tyres, which may not fully reflect the actual situation. The article also provides the comparison of measurement accuracy between the axle scales used by police and the pallet truck weighing scale. An innovation is to perform the experimental measurements in order to assess the impact of load distribution as well as the axle load and centre of gravity’s position on a vehicle’s ability to accelerate and on a vehicle’s tractive force intensity. This article brings the results covering several situations, such as the actual deformation of tyres, suspension, the actual position of load’s centre of gravity, weight, driver’s position and many other factors that are simplified or neglected with simulations, and these may be a significant practical and scientific contribution and innovation as well.

## Methodology

The purpose of measuring was to determine the change in a position of the centre of gravity of selected vehicles as a result of different load distribution. The measurements were performed with passenger vehicle, van and truck:

- Kia Ceed ECO Dynamics
- Citroën Jumper
- MAN 18.224 LLC

### Measurements with Kia Ceed ECO

The technical parameters of Kia Ceed ECO are given in Table 1.

**Figure 1:**
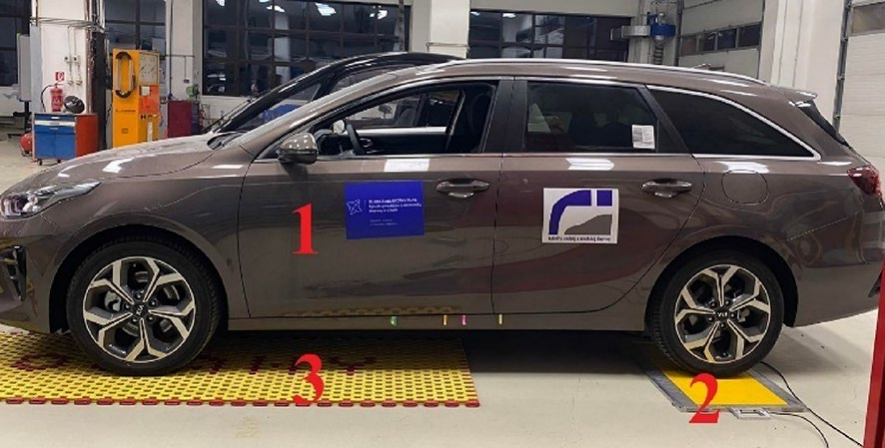
Principle of determining the axle load via portable weighing system: Tenzováhy PW-10.

The measurements were performed via a portable weighing system: Tenzováhy PW-10 (Dynamic and Static Scales), as also seen in Fig. 1, Position 2. The maximum permissible scale load per one wheel is 10,000 kg. It is 20,000 kg per axle, and one scale division is 20 kg and the deviation of weighing given by the manufacturer is 50 kg per axle^28^.

**Table 1:** Technical parameters of Kia Ceed ECO^27^.

Parameter	Value
Engine	1.5T-GDI GPF
Engine power, engine type	118 kW, spark-ignition
Outside size, body size (l/w/h)	4310/1800/1447 mm
Wheelbase	2650 mm
Total mass	1850 kg
Kerb mass	1425 kg
Load capacity	425 kg
Maximum permissible roof load	80 kg
Maximum front axle load	1200 kg
Maximum roar axle load	1150 kg

The vehicle (Fig. 1, Position 1) drove its rear axle onto the scales (2). To make the vehicle in the plane during the measurement, there were mats (3) given under the front axle, which were of the same height as the scales (2). When the weight applied to the rear axle was determined, the vehicle was turned so as the front axle was on the scales and the rear axle was on the carpet.

**Figure 2:**
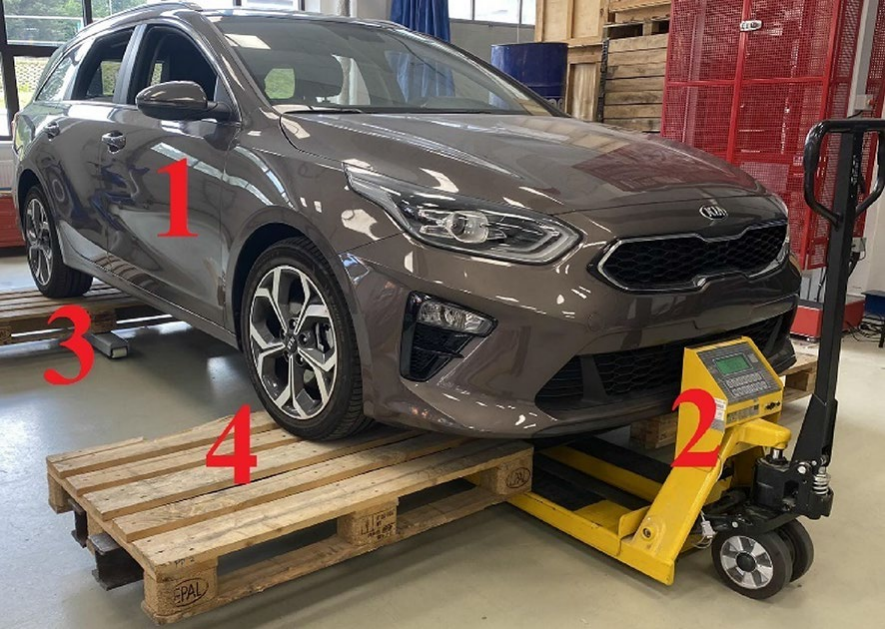
Principle of determining the axle load via the pallet truck weighing scale and pallet scales.

Since, according to the manufacturer, the deviation of weighing via the weighing system PW 10 is up to 50 kg, the measurements with Kia Ceed to determine the accuracy of weighing were also performed with the pallet truck weighing scale KPZ 52E-7/1 and the pallet scales LP7516 (Fig. 2). The scale division of both was 0.5 kg and the deviation of weighing was 0.5 kg^29,30^.

The vehicle (Position 1, Fig. 2) was placed on the pallets (Position 4). The pallet truck weighing scale was under the pallets of the front axle (Position 2) and the pallet scales were under the pallets of the rear axle (Position 3). The horizontality of both axles was provided by adjustment of the pallet truck weighing scale’s height. Thus, it was possible to compare the measurement accuracy between the axle scales with scale division of 20 kg and given weighing deviation of 50 kg per axle and the pallet truck weighing scale together with pallet scales, with measurement accuracy of 0.5 kg.

The measurements of the weight applied to the axles were performed as following:

- The empty vehicle with the driver only weighing 50.5 kg.
- Load placed in the luggage compartment weighing 210 kg.
- Load placed on the front seat and on the floor in front of the front seat weighing 210 kg.
- Load placed in the luggage compartment, and on the front seat and on the floor in front of the front seat weighing 420 kg.
- Load placed in the luggage compartment weighing 420 kg.

**Figure 3:**
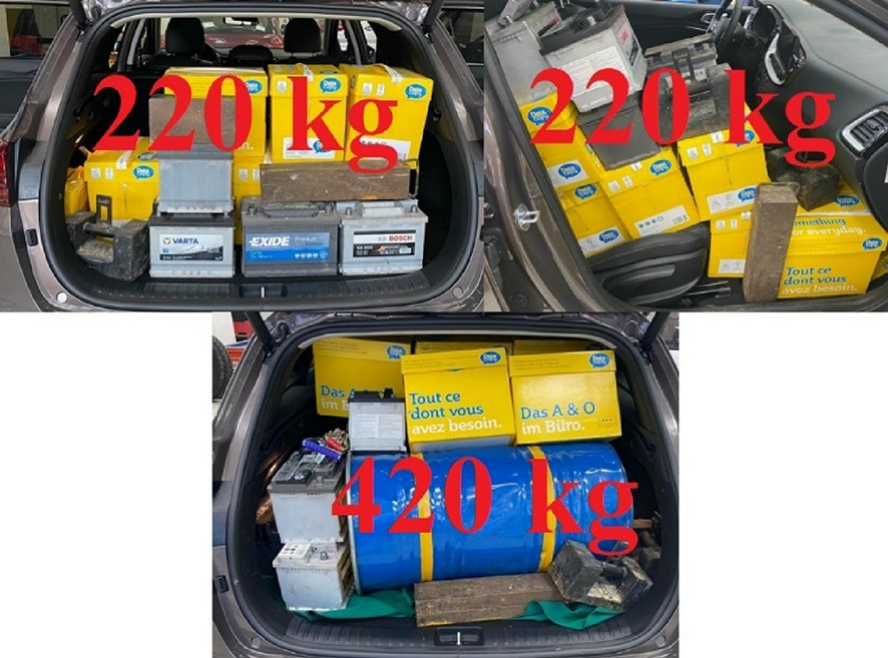
Load of Kia Ceed ECO.

Loads of 220 kg and of 420 kg are shown in Fig. 3.

The weight of vehicle load was determined by the pallet truck weighing scale KPZ 52E-7/1.

The distance of the centre of gravity from the front axle can be determined as:

1 $$ L_{1} = \frac{{m_{2} \cdot L}}{m} $$

where, L_1_ is the distance of the centre of gravity from the front axle [m], m_2_ is the weight applied to the rear axle [kg], L is the axle wheelbase [m], m is the vehicle total mass [kg].

The total mass was calculated by addition of the weights applied to the front and rear axle. Likewise it is also possible to calculate the distance of the centre of gravity from the rear axle as:

2 $$ L_{2} = \frac{{m_{1} \cdot L}}{m} $$

where, L_2_ is the distance of the centre of gravity from the front axle [m], m_1_ is the mass applied to the front axle [kg], L is the axle wheelbase [m], m is the vehicle total mass [kg]^31^.

To understand the impact of various load distribution on vehicle performances better, there were the measurements of vehicle tractive force performed at different load distribution. Kia Ceed was fixed to another vehicle by a chain. The chain had implemented a dynamometer Load-Cell-619 from which the data were processed via IPRE2 4S together with relevant software recording the force in the chain between vehicles, i.e. the tractive force of 100 Hz frequency. The measurement deviation of the dynamometer is max. 0.030% from the value measured^32^. The measurement was performed at the level ground on wet asphalt surface. The vehicle had summer tyres Michelin PRIMACY 4 225/45 R17 on with the depth of tyre tread of about 5 mm. The driver engaged the first gear and applied the acceleration pedal fully. Then the clutch pedal was released gradually until the full engagement. Since the vehicle was fixed to another vehicle by the chain with a dynamometer, it was possible to record the tractive force at various load distribution, and thus at various driving axle loads.

The Kia Ceed’s ability to accelerate uphill at various load distributions was determined as well. The measurement was performed on a wet asphalt surface with 6% gradient. The driver engaged the first gear and applied the acceleration pedal fully. Then the clutch pedal was released as fast as possible. The distance and time needed to reach the speed of 20 km·h^−1^ were recorded by XL Meter Pro Gamma Expert with the record frequency of 200 Hz and measurement deviation^33^ of 0.005 m·s^−2^.

### Measurements with Citroën Jumper

The measurements were performed via a portable weighing system: Tenzováhy PW-10—dynamic and static scales (Fig. 1, Position 2), like with Kia Ceed ECO. Technical parameters of Citroën Jumper are given in Table 2.

**Table 2:** Technical parameters of Citroën Jumper^34^.

Parameter	Value
Engine	2.2 l
Engine power, engine type	103 kW, compression ignition
Outside size, body size (l/w/h)	5998/2050/2524 mm
Wheelbase	4040 mm
Front/rear track	2060–2280 mm
Total mass	3500 kg
Operating mass	2167 kg
Load capacity	1333 kg
Maximum front axle load	1870 kg
Maximum rear axle load	2000 kg

**Figure 4:**
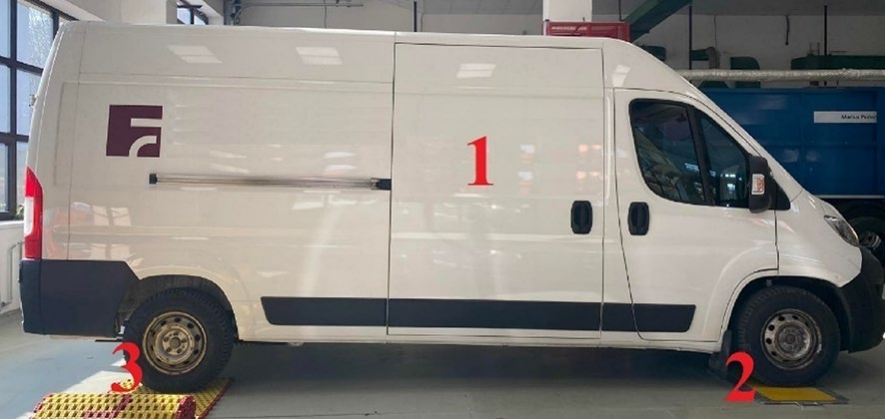
Citroën Jumper during measurements.

Citroen Jumper, which was used for measuring, is displayed in Fig. 4, position 1.

The measurements of the weight applied to the axles were performed as following:

- The empty vehicle with the driver only weighing 50.5 kg.
- Load placed in the front part of the load compartment, leaning against the front face of the load compartment, weighing 1000 kg.
- Load placed in the middle of the load compartment weighing 1000 kg.
- Load placed in the rear of the load compartment, leaning against the rear door of the load area, weighing 1000 kg.

**Figure 5:**
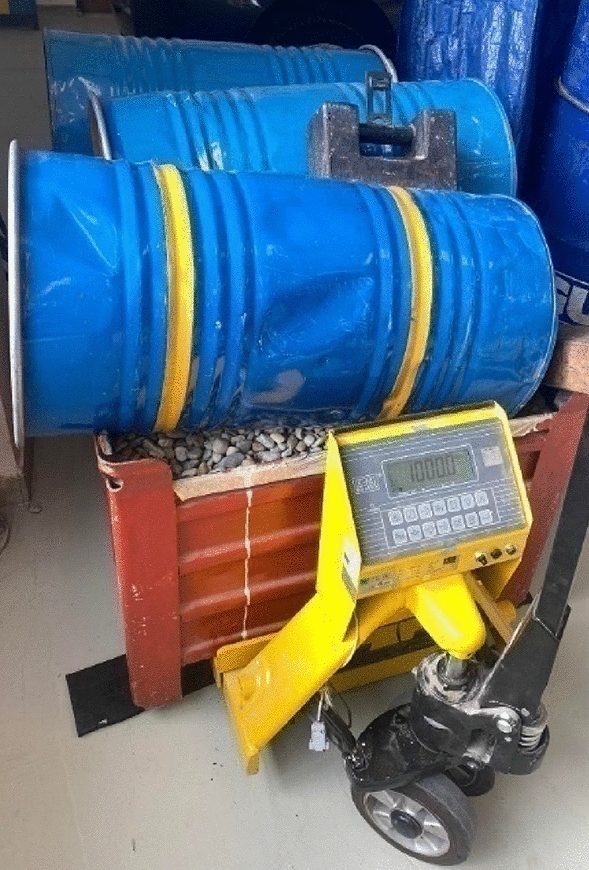
Load of Citroën Jumper.

Load of 1000 kg used when measuring with Citroën Jumper is shown in Fig. 5. The stack pallet is 800 mm wide and 1200 mm long.

The calculation of the centre of gravity’s distance from the front and rear axle was done by formula (1) and (2).

When the measurements with a load of 1000 kg were performed, the calculations were simulated to determine the change in the centre of gravity’s position in case of heavier load. The simulated load mass was determined by the maximum load capacity of Citroën Jumper (1260 kg), located in the same stack pallet. Since the load of Citroën Jumper was homogeneous (Fig. 5), it could be assumed the centre of gravity to be placed in its centre. The pallet was located longitudinally against the vehicle, so the centre of gravity was 600 mm from its start and 600 mm from its end as well. The calculation of the change in the centre of gravity’s position was also simulated when there was a longer version of Citroën Jumper marked as L4. The length of the vehicle would have been 6.363 m in this case, with the same wheelbase.

To calculate the centre of gravity’s distance of a loaded vehicle from the front axle when the load is heavier, or the vehicle is longer, the following formula was used:

3 $$ L_{1S} = \frac{{m_{vU } \cdot L_{1} + m_{L} \cdot (L - L_{SC2} )}}{{m_{Load} }} $$

where, L_1S_ is the centre of gravity’s distance of the loaded vehicle from the front axle determined by the simulation [m], m_vU_ is the mass of the unladen vehicle [kg], L_1_ is the distance of the centre of gravity of the empty vehicle from the front axle [m], L is the axle wheelbase [m], L_SC2_ is the distance of the centre of gravity of the load itself from the rear axle [m], m_Load_ is the total mass of the vehicle including the load [kg]^35^.

It was also a calculation for the distance of the centre of gravity of the fully loaded, or longer vehicle from the rear axle performed analogically.

### Measurements with MAN

The measurements with MAN focused on determining the change in the centre of gravity’s position in the plane due to different load distribution (Fig. 14). Technical parameters of MAN are given in Table 3.

**Table 3:** Technical parameters of MAN 18.224 LLC^36^.

Parameter	Value
Engine	6.9 l
Engine power, engine type	162 kW, compression ignition
Outside size, body size (l/w/h)	6550/2540/3200 mm
Wheelbase	3880 mm
Front/rear track	2060/1810 mm
Total mass	18,000 kg
Kerb mass	7705 kg
Load capacity	10,295 kg
Maximum front axle load	7100 kg
Maximum rear axle load	11,500 kg

**Figure 6:**
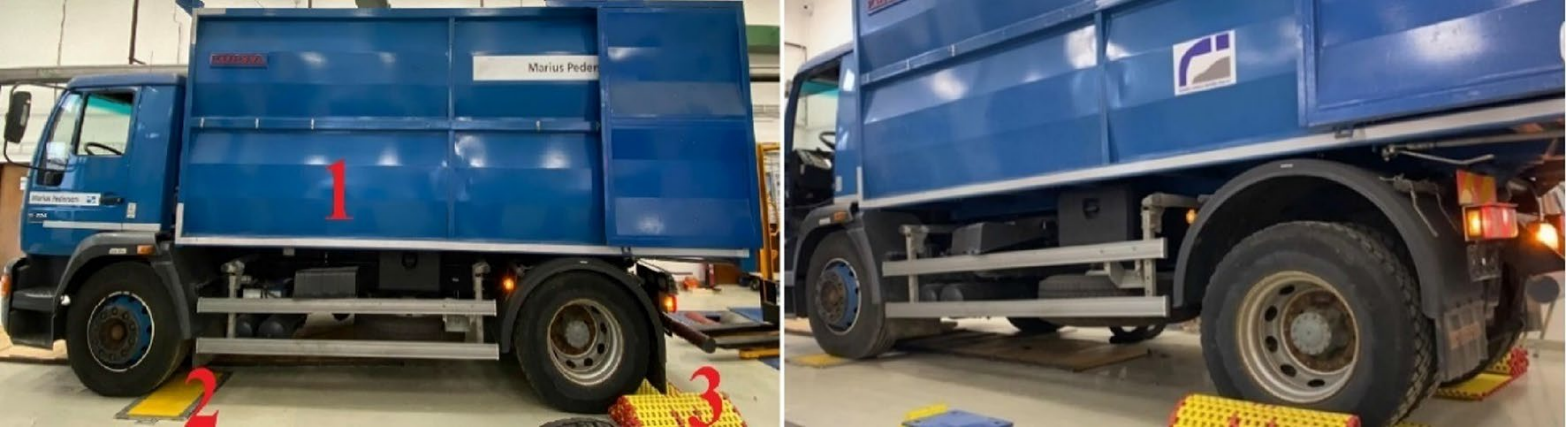
MAN during the measurements.

MAN, which was used for measuring, is displayed in Fig. 6. The measurements were performed in the plane (Fig. 6 in the left), and with lifted load area (Fig. 6 in the right).

The measurements were performed via a portable weighing system: Tenzováhy PW-10—Dynamic and Static Scales (Fig. 1, Position 2) like with Kia Ceed ECO.

The measurements of the weight applied to the axles were performed as following:

- The empty vehicle with the driver only weighing 50.5 kg.
- Load leaned against the front face of the load area weighing 2000 kg.
- Load in the middle of the load area weighing 2000 kg.
- Load leaned against the rear face of the load area weighing 2000 kg.

**Figure 7:**
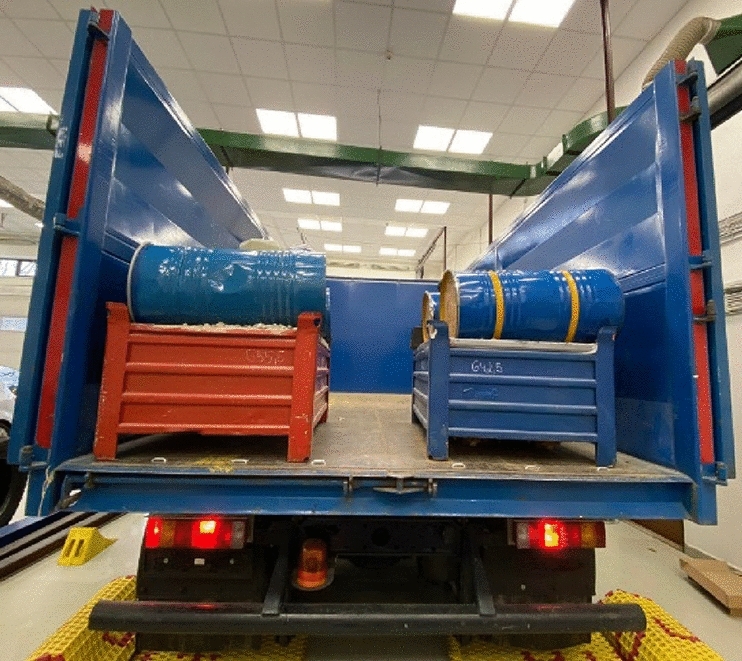
Load of MAN.

The load of MAN is shown in Fig. 7.

The position of the centre of gravity in the horizontal direction was calculated according to formulas (1) and (2). There was the mathematical simulation performed with MAN if the load mass would be of 4000 kg and of 6000 kg. The calculation was done by formula 3.

## Results

### Measurement results with Kia Ceed ECO

The measured values and measurement deviations are given in Table 4. The second and third column shows the value before the slash that is determined by the axle scales PW 10. At the same time, the second column shows the value after the slash that is determined by the pallet truck weighing scale, and the third column shows the value after the slash that is determined by the pallet scales. The weight of the load itself was determined by the pallet truck weighing scale with the measurement deviation of 0.5 kg. The fourth column shows the “Measured vehicle mass”, which means the weight of vehicle with load determined by the axle scales PW 10, and the fifth column shows the “Theoretical vehicle mass”, which means the sum of weights of the empty vehicle and the load itself determined by the pallet truck weighing scale. This is followed by the percentage difference between these two values, which can be considered as a percentage value of the weighing deviation when using the axle scales Tenzováhy PW 10.

**Table 4:** Measured values and measurement deviations of Kia Ceed ECO.

	Front axle load [kg]	Rear axle load [kg]	Measured vehicle mass [kg]	Theoretical vehicle mass [kg]	Deviation [%]
Empty vehicle	800/799.5	580/580.5	1380	1380	0.00
Load in the luggage compartment, 210 kg	740/743	860/847	1600	1590	+ 0.63
Load on the front seat and in front of it, 210 kg	920/926	660/664	1580	1590	− 0.53
Load in the luggage compartment, 210 kg, and on the front seat and in front of it, 210 kg	900/890.5	920/909	1820	1800	+ 1.10
Load in the luggage compartment, 420 kg	720/719	1080/1081	1800	1800	0.00

As follows from Table 4, load distribution in a passenger car has a large impact on the axle load. Comparison of the values determined by the axle scales PW 10 (second and third column before the slash) and the values determined by the pallet scales, or the pallet truck weighing scale (second and third column after the slash) has shown the maximum deviation of 11 kg per axle in relation to “load in the luggage compartment, 210 kg, and on the front seat and in front of it, 210 kg”. The deviations observed have never reached the deviations of 50 kg given by the manufacturer. The reason is probably the fact that the axle scales PW 10 are almost exclusively used by police to determine the weights of heavy vehicle’s axles in the field. Police usually performs the measurements directly on the road which does not need to be at exact level ground, and the engine is usually working, so the weight of axles is determined in motion while driving slowly on the scales. Since police may sanction on the basis of weights applied to each axle, the manufacturer has given the deviation up to 50 kg per axle^37^. However, this deviation could have been significantly reduced by weighing in the laboratory at the level ground with a stopped engine and without any motion, as also follows from Table 4. Weighing with the pallet truck weighing scale and the pallet scales is time-consuming many times and cannot be used for heavier vehicles, therefore, when assessing the real deviation of the axle scales, weighing with the axle scales PW 10 is considered appropriate and sufficiently accurate in this case.

Comparison of the weight determined by the axle scales PW 10 and the weight calculated by weighing the load itself via the pallet truck weighing scale shows that the largest deviation was 1.10%, resp. 20 kg (Table 4, last column). Such deviation can be considered as a sufficiently accurate measurement.

For better understanding, the division of vehicle mass into the front and rear axles is displayed in percentage in Table 5. Table 5 also shows the calculated percentage change in the axle load in comparison with the empty vehicle and the distance of the centre of gravity from the front and rear axles.

**Table 5:** Division of vehicle mass into the front and rear axles of Kia Ceed ECO.

	Front axle load [%]	Rear axle load [%]	Change [%]	L1 [m]	L2 [m]	Change [m]
Empty vehicle	58	42	0	1.12	1.53	0
Load in the luggage compartment, 210 kg	46	54	12	1.43	1.22	0.31
Load on the front seat and in front of it, 210 kg	58	42	0	1.11	1.54	-0.01
Load in the luggage compartment, 210 kg, and on the front seat, 210 kg	49	51	9	1.34	1.31	0.22
Load in the luggage compartment, 420 kg	40	60	18	1.59	1.06	0.47

The empty vehicle, only with the driver weighing 50.5 kg and the vehicle with the load weighing 210 kg, which was on the front seat and on the floor in front of it, had the same percentage axle load. This situation could occur when the centre of gravity of the load was placed in the centre of gravity of the empty vehicle, as also follows from formula (3). Thus, loading the load of 210 kg on the front seat and on the floor in front of did not lead to a shift of the centre of gravity in the horizontal direction.

The distance of the centre of gravity with a load of 420 kg shifted by 47 cm from the front axle to the rear one (Table 5).

Table 6 serves for better display of the impact of load distribution in a passenger car on selected vehicle parameters. The second column of Table 6 shows the tractive force reached by the vehicle on wet asphalt surface. The third column shows the percentage change in comparison with the empty vehicle. The fourth column shows the time and the fifth column shows the distance needed to reach the speed of 20 km·h^−1^ on wet asphalt surface with 6% gradient. The last column shows the change of needed distance in comparison with the empty vehicle.

**Table 6:** Measured values and measurement deviations of Kia Ceed ECO.

	Tractive force [daN]	Change [%]	Time [s]	Distance [m]	Change [%]
Empty vehicle	708	0	1.97	5.67	0
Load in the luggage compartment, 210 kg	506	− 28	2.80	8.00	41
Load on the front seat and in front of it, 210 kg	840	18	2.16	6.60	16
Load in the luggage compartment, 210 kg, and on the front seat and in front of it, 210 kg	851	20	2.16	6.60	16
Load in the luggage compartment, 420 kg	505	− 28	3.10	8.60	52

As follows from data in Table 6, cargo load and its distribution in the vehicle can significantly affect road safety. When the load of 210 kg is placed in the luggage compartment, the vehicle tractive force decreased by 28% due to reduced load on the front axle, and the distance needed to reach the speed of 20 km·h^−1^ increased by up to 41%. When the load with the same weight was placed on the front seat and in front of it, the tractive force had increased by 18%, in comparison with the empty vehicle, and the distance needed for starting the vehicle had lengthened only by 16%. The reason why the length of the distance got longer even though the tractive force was higher is that both gradient and inertia resistances had increased as well^38,39^. When the load of 420 kg was placed in the luggage compartment, the tractive force had decreased by 28% in comparison with the empty vehicle, and the distance needed for starting the vehicle had lengthened by 52%. Such an increase in time and distance needed for a vehicle’s acceleration may be crucial when car accidents happen, for instance when driving from the side road to the main road since there is a higher need to accelerate^40^. The results highlight the importance of paying attention to the centre of gravity in relation to axles, i.e. in the horizontal direction.

The experimental measurement also proved that the tractive force does not increase exactly linearly with the axle load, but it is also affected by ESP, ASR, size of contact area between the tyre and road surface, tyre deformation and by many other factors that are included in the experimental measurements as opposed to the simulations^41–43^.

**Figure 8:**
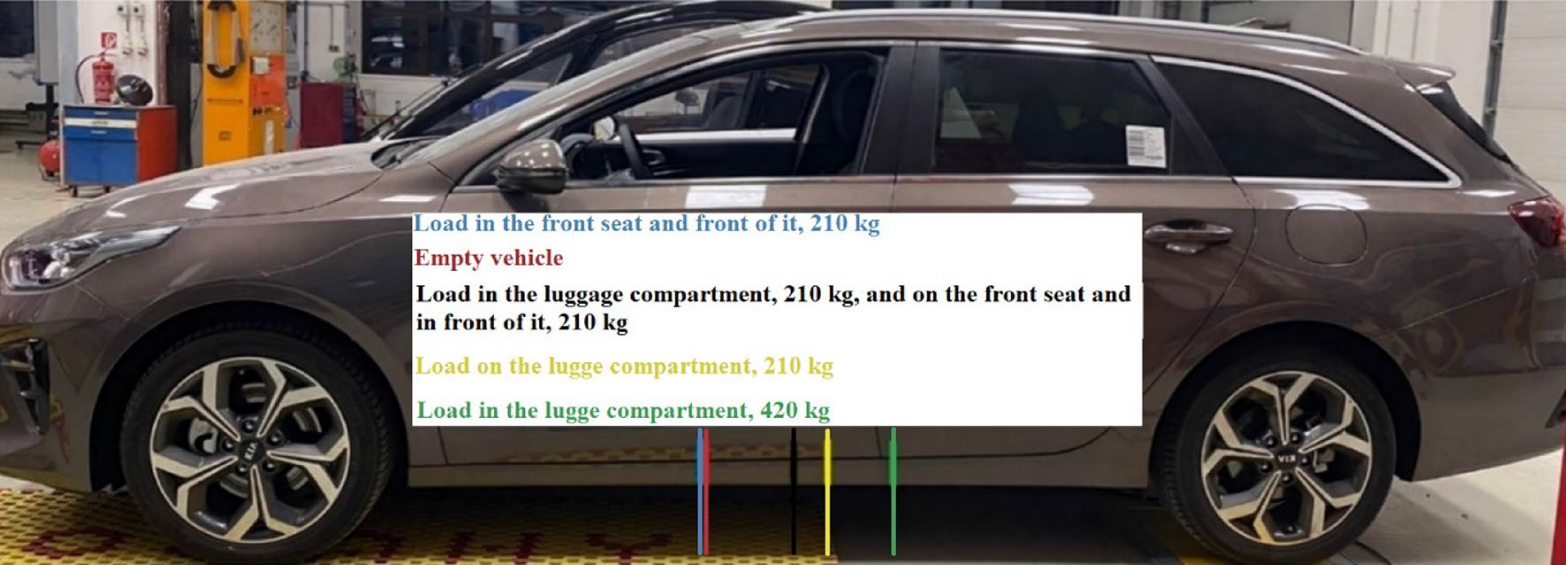
Position of the centre of gravity in a horizontal direction of Kia Ceed ECO.

The position of the Kia Ceed ECO’s centre of gravity in a horizontal direction is displayed in Fig. 8. Each variant of load position has its own colour markings.

The load weight of 420 kg represents 89% of the vehicle load capacity. The rear axle was not overloaded, however, the centre of gravity shifted rearwards substantially, as shown in Fig. 8 as well. Such change in the position of the centre of gravity also affects the vehicle driving performances, due to change in the suspension performances as well, which is also mentioned in publication^44^. There is lower weight applied to the front axle, i.e. 720 kg from the vehicle weighing 1800 kg, which is 40%. Thus, the front driving axle can shift the lower driving force onto the road, which may decrease the vehicle’s ability to accelerate in combination with higher vehicle mass or icy road. Reducing the load of the front axle increases the risk of an understeer skid, which means that the vehicle’s ability to bend is reduced.

### Measurement results with Citroën Jumper

The measurements were performed with the axle scales with the load of 1000 kg placed in the load compartment in front, in the middle of the load compartment and in its rear. Table 7 shows the values measured according to formulas (1) and (3) for simulation of a longer version of the vehicle and the load with the weight equals to the load capacity. To verify the measurement accuracy, Table 7 shows the measurement deviations, too.

**Table 7:** Measured and calculated data with deviations of Citroën Jumper.

	Front axle load [kg]	Rear axle load [kg]	Measured vehicle mass [kg]	Theoretical vehicle mass [kg]	Deviation [%]
Empty vehicle	1420	820	2240	2240	0.00
Load placed in the front, 1000 kg	1940	1340	3280	3240	+ 1.21
Load placed in the middle, 1000 kg	1680	1600	3280	3240	+ 1.21
Load placed in the rear, 1000 kg	1420	1880	3300	3240	+ 1.82
Load placed in the rear, 1000 kg, longer vehicle	1413	1867	–	–	–
Load placed in the front, 1260 kg	2140	1360	–	–	–
Load placed in the middle, 1260 kg	1603	1897	–	–	–
Load placed in the rear, 1260 kg	1421	2079	–	–	–

At the stowage of the load of 1000 kg, which is 79% of the load capacity, the maximum load on the front axle was exceeded when the load was in the front. The front axle was overloaded by 70 kg. Concerning the simulation with the longer vehicle and the load located in the rear, the weight applied to the rear axle did not exceed, and the weight applied to the front axle did not decrease under 25% from the total vehicle mass, thus the minimum steering axle load given by the legislation was not exceeded. When simulating the load capacity used to 100% and the load placed in the front, the front axle was overloaded by 270 kg, which is 14.44%. When the load is placed in the rear, the rear axle was overloaded by 79%, which is 3.95%. The only way to stowage the load without any risk of axle overload is to place the load in the middle of the vehicle load area. However, this is not preferred to the other two ways of stowage. When the load is in the rear, it can be loaded and unloaded easily via forklift. When the load is in the front, it can be lean against the front face of the load area, and thus, to reduce the number of binding straps. When the load is in the middle of the load area, it is not possible to use any of the above mentioned advantages, so it is not often used^45^. The measurement deviation with Citroën Jumper reached up to 60 kg, which was 1.82% from the mass. The distance of the centre of gravity from the front and rear axles, with various load stowage and simulation of the longer vehicle and heavier load, are given in Table 7, too.

The proportional division of the vehicle mass between the front and rear axle is given in Table 8.

**Table 8:** Division of the vehicle mass into the front and rear axle of Citroën Jumper.

	Front axle load [%]	Rear axle load [%]	Change [%]	L1 [m]	L2 [m]	Change [m]
Empty vehicle	63	37	0	1.48	2.56	0
Load placed in the front, 1000 kg	59	41	4	1.65	2.39	0.17
Load placed in the middle, 1000 kg	51	49	12	1.97	2.07	0.49
Load placed in the rear, 1000 kg	43	57	20	2.30	1.74	0.82
Load placed in the rear, 1000 kg, longer vehicle	43	57	20	2.34	1.7	0.86
Load placed in the front, 1260 kg	61	39	2	1.57	2.47	0.09
Load placed in the middle, 1260 kg	46	54	17	2.19	1.85	0.71
Load placed in the rear, 1260 kg	41	59	22	2.40	1.64	0.92

The load of 1260 kg caused a higher difference in the axle load than the simulation of a longer vehicle version, as follows from Table 8.

When simulating the load of 1260 kg placed rear, it led to a change in the position of the centre of gravity even by 92 cm, and substantial changes in the vehicle driving performance might be assumed, especially in the vehicle’s ability to bend and accelerate, and to decelerate, as also mentioned in publication^35^ in which the research focused on the impact of load of a van for vehicle transportation on its ability to accelerate and decelerate. The results from publication^46^ affirm the need to consider the change in the vehicle performances due to its load since loading the van led to an increase in time needed for acceleration, and to a decrease in braking deceleration. The authors^46^ pointed to the change in tyre characteristics depending on their load as well.

**Figure 9:**
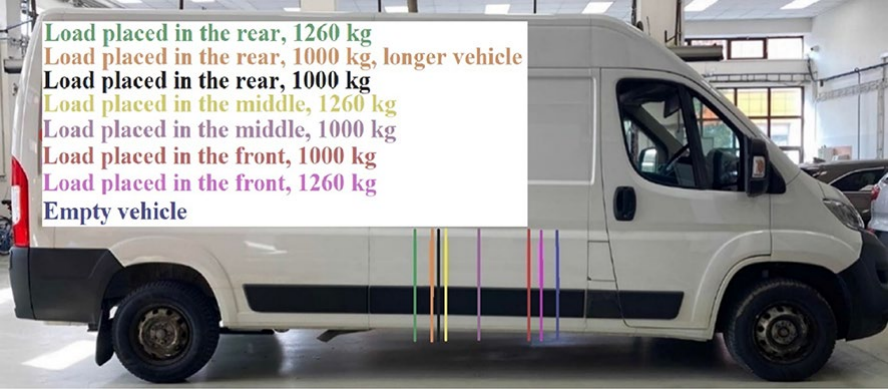
Position of the centre of gravity in the horizontal direction of Citroën Jumper.

Placing of the centre of gravity displayed on the vehicle is given in Fig. 9.

The position of the centre of gravity in the horizontal direction was changing due to the load shifting as well as different load weight, and it had an impact on risk of the axle overload. Relating a van, it is necessary to take into consideration the risk of overloading the front axle when distributing the load.

### Measurement results with MAN

The measurements with MAN focused on determining the position of the centre of gravity in the horizontal direction with the load area in the level position and with the lifted load area above the rear axle. Heavier loads were also simulated. The results measured with the axle scales and the results achieved by the simulation are given in Table 9. The weighing deviations of axle scales are given in Table 9, too.

**Table 9:** Measured and calculated data with deviations of MAN.

	Front axle load [kg]	Rear axle load [kg]	Measured vehicle mass [kg]	Theoretical vehicle mass [kg]	Deviation [%]
Empty vehicle	4000	3840	7840	7840	0.00
Load placed in the front, 2000 kg	5400	4440	9840	9840	0.00
Load placed in the middle, 2000 kg	4500	5360	9860	9840	+ 0.20
Load placed in the rear, 2000 kg	3640	6140	9780	9840	− 0.61
Load placed in the front, lifted load area, 2000 kg	5480	4340	9820	9840	− 0.20
Load placed in the rear, lifted load area, 2000 kg	3720	6060	9780	9840	− 0.61
Load placed in the front, 4000 kg	6804	5036	–	–	–
Load placed in the rear, 4000 kg	3357	8483	–	–	–
Load placed in the front, 4000 kg	8811	5029	–	–	–
Load placed in the rear, 6000 kg	3032	10,808	–	–	–

Likewise in the Citroën Jumper, the front axle of MAN was overloaded without using the load capacity completely. The front axle was overloaded by 24% when using the load capacity to 58% (i.e. the load mass was 6000 kg and the load capacity was 10,295 kg). The cause was that the load was placed in front of the load area since the weight applied to the front axle with the empty vehicle is 4000 kg, and maximum permissible weight of the front axle is only by 3100 kg higher. Lifting of the load area above the rear axle caused the reduction of the load of the rear axle by 80 kg, and the front axle increased by the same weight. Lifting of the rear axle with heavier load would have had an impact on the measurement results at the road inspection in some cases. The maximum deviation when weighing was 60 kg, however, it was expressed as 0.60% percentage. Thus, the deviations are lower than with Citroën Jumper.

The proportional division of the mass into the front and rear axle is given in Table 10. Table 10 shows the distance of the centre of gravity in the horizontal direction from the front and rear axles, too. It also displays the shift of the centre of gravity in comparison with the empty vehicle due to load stowage.

**Table 10:** Division of the vehicle mass into the front and rear axle of MAN.

	Front axle load [%]	Rear axle load [%]	Change [%]	L1 [m]	L2 [m]	Change [m]
Empty vehicle	51	49	0	1.90	1.98	0
Load placed in the front, 2000 kg	55	45	4	1.75	2.13	0.15
Load placed in the middle, 2000 kg	46	54	5	2.11	1.77	0.21
Load placed in the rear, 2000 kg	37	63	14	2.44	1.44	0.54
Load placed in the front, lifted load area, 2000 kg	56	44	5	1.71	2.17	0.19
Load placed in the rear, lifted load area, 2000 kg	38	62	13	2.40	1.48	0.5
Load placed in the front, 4000 kg	57	43	6	1.65	2.23	0.25
Load placed in the rear, 4000 kg	28	72	23	2.43	1.45	0.53
Load placed in the front, 6000 kg	64	36	13	1.41	2.47	0.49
Load placed in the rear, 6000 kg	22	78	29	3.03	0.85	1.13

The stowage of the load of 6000 kg to the rear, for instance due to easier loading and unloading, would reduce the load of the front steering axe, so as it would transfer only 22% of the vehicle mass. This would be dangerous since the vehicle would be susceptible to the oversteer skid. On the other hand, a larger load on the driving axle is required when the vehicle needs to start driving uphill on the surface with a low friction coefficient, for example on ice, loam or sand^47^ if there is no wheel sinking into the mat. An ideal distribution of the weight 50 to 50 is when the vehicle is empty.

**Figure 10:**
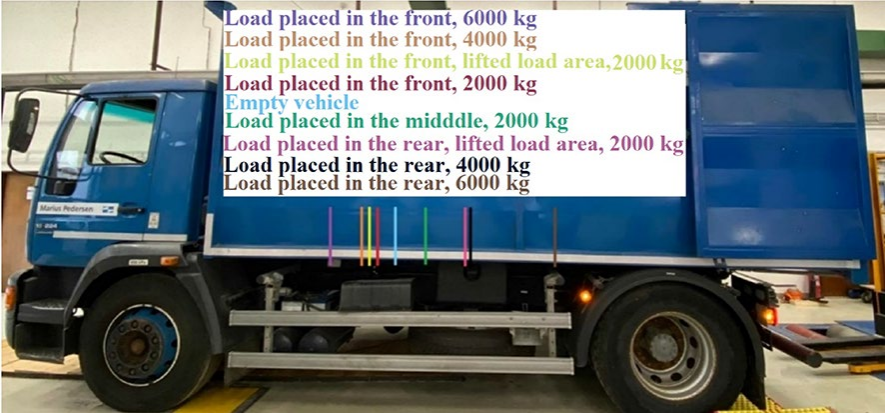
Position of the centre of gravity in a horizontal direction of MAN.

Concerning the empty vehicle, the centre of gravity was approximately in the middle between the front and rear axle, which is an ideal position theoretically. To keep this position of the load, it would be necessary to place the load in a way in which the centre of gravity would be located in the centre of gravity of the empty vehicle. Graphical display of the location of the centre of gravity in a horizontal direction is given in Fig. 10.

MAN has a relatively short wheelbase (3.88 m), and it is shorter than the wheelbase of Citroën Jumper (4.04 m). The rear overhang of MAN is only 1.2 m. The combination of such a short wheelbase and short rear overhang enables more variable load distribution. In case of long wheelbase and long rear overhang, there would be a risk of overload or too reduced load of any axles substantially higher.

## Conclusion

The main research question was the extent of the impact of load distribution on the axle load in selected vehicle categories on the change of the centre of gravity’s position and the axle load as factors affecting the road safety. In order to answer the question, there were extensive experimental measurements performed under laboratory conditions with three different vehicles. Based on the results with the passenger vehicle, it can be concluded low risk of axle overload due to loads. The risk in relation to passenger cars is reducing the load of the front steering and also the driving axle, especially with vehicles with longer rear overhang which is typical for an estate car body-style. It can result in so-called phantom congestion, especially for a number of junctions in succession, which is a frequent occurrence.

Concerning the VAN vehicles, it is necessary to place the load on a given position since there is a risk of overload, mainly of the front axle, even when not using the vehicle load capacity completely. A similar situation was proved with a truck as well. In relation to a van, especially its longer version, there is also a risk of reducing the load of the front axle. There is a need to pay attention to place the load properly also if the load capacity is not used completely when loading as well as when unloading gradually. The axle overload if the vehicle total mass is not exceeded is also connected with a practice when only the total mass of vehicles is measured in the undertakings, not the mass applied to each axle.

As also mentioned in the introduction of this study, the roads experience the issue of ruts as a result of axle load of the single tyres. The measurements with MAN have shown that the axle overload can also occur even if the vehicle load capacity is not used completely when the load is not distributed correctly. On the other hand, when the load is distributed correctly, the load capacity can be used completely, also in the case of a van, without any axle overload.

The study also focused on the question to what extent can the value of a vehicle’s tractive force and ability to accelerate be affected by various load distributions. The experimental measurements were performed with a passenger car. The measurement proved that there is not any exact linear dependence between the weight applied on the axle and the tractive force, but it is necessary to take into consideration many other factors in the case of simulations. Based on the results of experimental measurements, it can also be concluded a large impact of load distribution on the vehicle’s ability to accelerate uphill. Besides the impact of axle load, the impact of inertia resistance and gradient resistance evinced during the measurements and measurement result analysis as well. What is more, it may be assumed that if there is 6% gradient when measuring, both reduction of the front driving axle load and the rear axle load increase and there will be some impact of the vehicle’s suspension and silencing. These and the other factors, such as vehicle electronic systems like ASR, are needed to be included into calculations when using the method of simulations in order to achieve the results as accurate as possible. For these simulations, it is also possible to use the results from this study either for comparison or as input data since all the factors and impacts were included in the experimental measurements.

Therefore, improper load distribution in any type of a vehicle can be the risk to road safety, and it can reduce the road lifespan, increase the number of congestion, PM in the air as well as the costs of transportation and vehicle services. On the contrary, when the load is distributed properly, the risk can be reduced substantially, the use of load capacity can be increased, and the number of driving can be reduced, which may contribute to reducing any adverse effects of the road transport on the human population.

The subsidiary research question concerned the possibility of using the axle scales for the measurements which aim to determine the axle load and to calculate the centre of gravity’s position in the horizontal direction. Based on the results of experimental measurements it can be concluded that this methodology is sufficiently accurate for determining the centre of gravity in the horizontal direction, since the largest theoretical measurement error reached 1.10% in the passenger car even when checked by weighing with the pallet scales and pallet truck weighing scale with the scale division of 0.5 kg. The measurement deviation of 50 kg per axle given by the manufacturer did not evince given the measurement conditions. Thus, the deviation of 50 kg per axle is relevant only when weighing on the road with uneven surface and when the vehicle is in motion. The largest deviation observed was 1.82% with VAN vehicle, when comparing the total vehicle mass and the weight of the load itself. Using the axle scales PW 10 would not be suitable for the measurements to determine the centre of gravity’s height since there will be large inaccuracies due to their measurement deviations. The above-mentioned deviation, which is less than 2%, is acceptable for the calculation of the vehicles’ centre of gravity in the horizontal direction, even when comparing the deviations occurring in simulations. Thus, data gained by the axle scales can also be used for the simulations, either as input data or for comparing the values observed, which may be considered as a significant contribution of this manuscript.

The result analysis of the experimental measurements has highlighted their advantage of covering several factors in comparison with methods of simulation. On the other hand, the disadvantage of experimental measurements is their extensive elaboration; they are time-consuming and place higher demands on the measurement technology. Application of the experimental measurement results is limited by the measurement deviations. It is necessary to pay attention to the measurement methodology when applying the results for calculations of the centre of gravity’s height in order to substantially reduce the measurement deviation aiming to have the results more accurate with a widespread use.

In the future, attention will also be paid to ensuring that the measurements shall be less labour intensive and time-consuming, which will reduce the disadvantages compared to simulations.

## Data Availability

All data generated or analyzed during this study are included in this published article.
